# Qualitative and Quantitative Requirements for Assessing Prognostic Markers in Prostate Cancer

**DOI:** 10.3390/microarrays3020137

**Published:** 2014-04-17

**Authors:** Christoph Burdelski, Aleksandra Matuszewska, Martina Kluth, Christina Koop, Katharina Grupp, Stefan Steurer, Corinna Wittmer, Sarah Minner, Maria Christina Tsourlakis, Guido Sauter, Thorsten Schlomm, Ronald Simon

**Affiliations:** 1 Institute of Pathology, University Medical Center Hamburg-Eppendorf, Martinistr. 52, 20246, Hamburg, Germany; E-Mails: cburdelski@uke.de (C.B.); matuszewska.aleksandra@yahoo.de (AM); m.kluth@uke.de (M.K.); c.koop@uke.de (C.K.); s.steurer@uke.de (S.S.); c.wittmer@uke.de (C.W.); s.minner@uke.de (S.M.); m.tsourlakis@uke.de (M.C.T.); g.sauter@uke.de (G.S.); 2 General, Visceral and Thoracic Surgery Department and Clinic, University Medical Center Hamburg-Eppendorf, Martinistr. 52, 20246, Hamburg, Germany; E-Mail: k.grupp@uke.de; 3 Martini-Clinic, Prostate Cancer Center, Martinistr. 52, 20246, Hamburg, Germany; E-Mail: tschlomm@uke.de; 4 Department of Urology, Section for Translational Prostate Cancer Research, University Medical Center Hamburg-Eppendorf, Martinistr. 52, 20246, Hamburg, Germany

**Keywords:** tissue microarray, prostate cancer, tissue quality, number of samples, prognosis, marker validation

## Abstract

Molecular prognostic markers are urgently needed in order to improve therapy decisions in prostate cancer. To better understand the requirements for biomarker studies, we re-analyzed prostate cancer tissue microarray immunohistochemistry (IHC) data from 39 prognosis markers in subsets of 50 – >10,000 tumors. We found a strong association between the “prognostic power” of individual markers and the number of tissues that should be minimally included in such studies. The prognostic relevance of more than 90% of the 39 IHC markers could be detected if ≥6400 tissue samples were analyzed. Studying markers of tissue quality, including immunohistochemistry of ets-related gene (ERG) and vimentin, and *fluorescence in-situ hybridization* analysis of human epidermal growth factor receptor 2 (HER2), we found that 18% of tissues in our tissue microarray (TMA) showed signs of reduced tissue preservation and limited immunoreactivity. Comparing the results of Kaplan-Meier survival analyses or associations to ERG immunohistochemistry in subsets of tumors with and without exclusion of these defective tissues did not reveal statistically relevant differences. In summary, our study demonstrates that TMA-based marker validation studies using biochemical recurrence as an endpoint require at least 6400 individual tissue samples for establishing statistically relevant associations between the expression of molecular markers and patient outcome if weak to moderate prognosticators should also be reliably identified.

## 1. Introduction

Prostate cancer is the most frequent malignancy in men. The clinical behavior ranges from slowly growing indolent tumors to highly aggressive and metastatic cancers. Based on the results of large autopsy studies demonstrating a high prevalence of prostate cancers also in men who never experienced symptoms of the disease during their lifetime, it is assumed that most prostate cancer patients would be manageable without surgery and its associated side effects [1]. Accordingly, distinguishing between the low malignant and indolent form of the disease that does not require immediate therapy and the aggressive cancers that will eventually progress to life-threatening disease is the clinically most relevant discipline of current prostate cancer research. 

Only recently, commercial molecular classifiers have become available. These tests are based on mRNA expression profiling of defined gene sets, allow for estimating the biological aggressiveness of a cancer and, therefore, may aid in therapy decisions [2,3,4]. These classifiers underscore the interest of the diagnostic industry in the topic of prostate cancer prognosticators. It can be expected that future generations of such classifiers can be substantially improved if genes are included that on their own already exhibit strong and independent prognostic power. 

During the last decades, a multitude of studies announced prognostic biomarkers for prostate cancer. However, although more than hundred different prognostic markers have been suggested (reviewed in [5]), none of them has entered clinical routine testing as to yet. This disappointing failure to translate research findings into clinical applications is partly due to the fact that data obtained on virtually all of these markers vary largely between different studies. This is even true for the most established prognostic parameters, such as p53 or phosphatase and tensin homolog (PTEN). More than 50 studies analyzed the impact of p53 alterations on prostate cancer phenotype and prognosis. Although most immunohistochemistry studies reported a link between nuclear p53 accumulation and adverse tumor features, such as high grade, advanced stage, and peripheral zone origin [6], as well as poor prognosis after radical prostatectomy [7] or external beam radiation [8] and unfavorable clinical courses in conservatively managed patients [9], there are also studies that do not confirm these associations [10,11]. Likewise, genomic deletion of PTEN has been unequivocally linked to adverse tumor features in several studies [12,13,14,15,16], other studies again employing immunohistochemistry reported highly variable results on the prognostic value of PTEN expression. For example, an association between loss of PTEN expression and poor patient prognosis was only found in one [13] out of four studies [13,17,18,19], and a link between loss of PTEN expression and high Gleason grade or advanced tumor stage was only reported in two [20,21] out of five studies on this topic [18,19,20,21,22]. 

It is quite obvious that most of the discrepant results in the literature are due to (i) technical issues, and (ii) relatively small patient cohorts used for most studies. It is obvious that different antibodies, staining protocols, and scoring criteria that are employed in most studies can cause massive experimental variation. Due to an intense dispute with a reviewer of one of our manuscripts on the issue whether our frequency of p53 immunostaining in prostate cancer was lower than the 50% suggested by another group due to protocol issues (in our opinion) or to missed heterogeneity in a tissue microarray (TMA) setting (the reviewer’s opinion), we were forced to experimentally demonstrate that the range of p53 positive prostate cancers could be brought from 2.5% to 98% solely by protocol modifications [7]. However, the example of HER2 immunohistochemistry analysis of breast cancer demonstrates that a considerable (but not a complete) degree of assay standardization can be achieved [23]. However, even in such a highly standardized analysis including various controls, the quality of the tissues samples will impact the results. This is due to the fact that postsurgical tissue fixation cannot be fully standardized. The most frequently used fixative, *i.e.*, formalin, causes proteins to cross-link and makes them impassible for microbial degradation or autolysis. The efficiency of the fixation process depends on the proper penetration of the formalin into the tissue, but obviously, the success of penetration greatly depends on the size and the composition of a given tissue. In case of too much or too little fixation, the tissue may not be suitable for analysis. This is a serious problem particularly in immunohistochemistry studies, where lack of immunoreactivity cannot be distinguished from a true negative result due to biological absence of the protein of interest. 

The tissue microarray (TMA) technology has proven to be excellently suited for rapid and cost efficient analysis of large numbers of tissue samples [24]. While in studies analyzing conventional large sections the study cohort size was typically limited to less than 100 samples due to the time and costs connected with such “classical” analyses, it is the availability of suitable tissues that first of all limits the size of TMA studies. As a consequence, TMA studies including hundreds of tissue samples are often viewed as “large-scale” analyses. Extent and impact of low-quality tissues that are inevitably included in every large-scale tissue analysis are, however, unknown. In the present study, we took advantage of our very large prostate cancer tissue microarray comprising more than 12,000 tissue spots and molecular data from more than one hundred proteins analyzed by means of immunohistochemistry to better understand the impact of the sample size and the tissue quality on the outcome of TMA studies for marker validation purposes. Biochemical (PSA) recurrence was used as an endpoint in this project dealing with patients having undergone prostatectomy. This reason for this is that PSA recurrence is the “easiest” (most frequent) clinical endpoint to analyze in prostatectomy studies and it is strongly associated with other clinical endpoints such as metastasis or cancer-related death.

## 2. Experimental Section

**Patients.** Radical prostatectomy specimens were available from 12,427 patients undergoing surgery between 1992 and 2012 at the Department of Urology and the Martini Clinics at the University Medical Center Hamburg-Eppendorf. Follow-up data were available for a total of 12,344 patients with a median follow-up of 36 months (range of 1 to 241 months; Table 1). Prostate specific antigen (PSA) values were measured following surgery and PSA recurrence was defined as a postoperative PSA of 0.2 ng/mL and increasing at first of appearance. All prostate specimens were analyzed according to a standard procedure, including a complete embedding of the entire prostate for histological analysis [7]. The TMA manufacturing process was described earlier in detail [24]. In short, one 0.6 mm core was taken from a representative tissue block from each patient. The tissues were distributed among 27 TMA blocks, each containing 144 to 522 tumor samples. For internal controls, each TMA block also contained various control tissues, including normal prostate tissue. 

**Figure 1:**
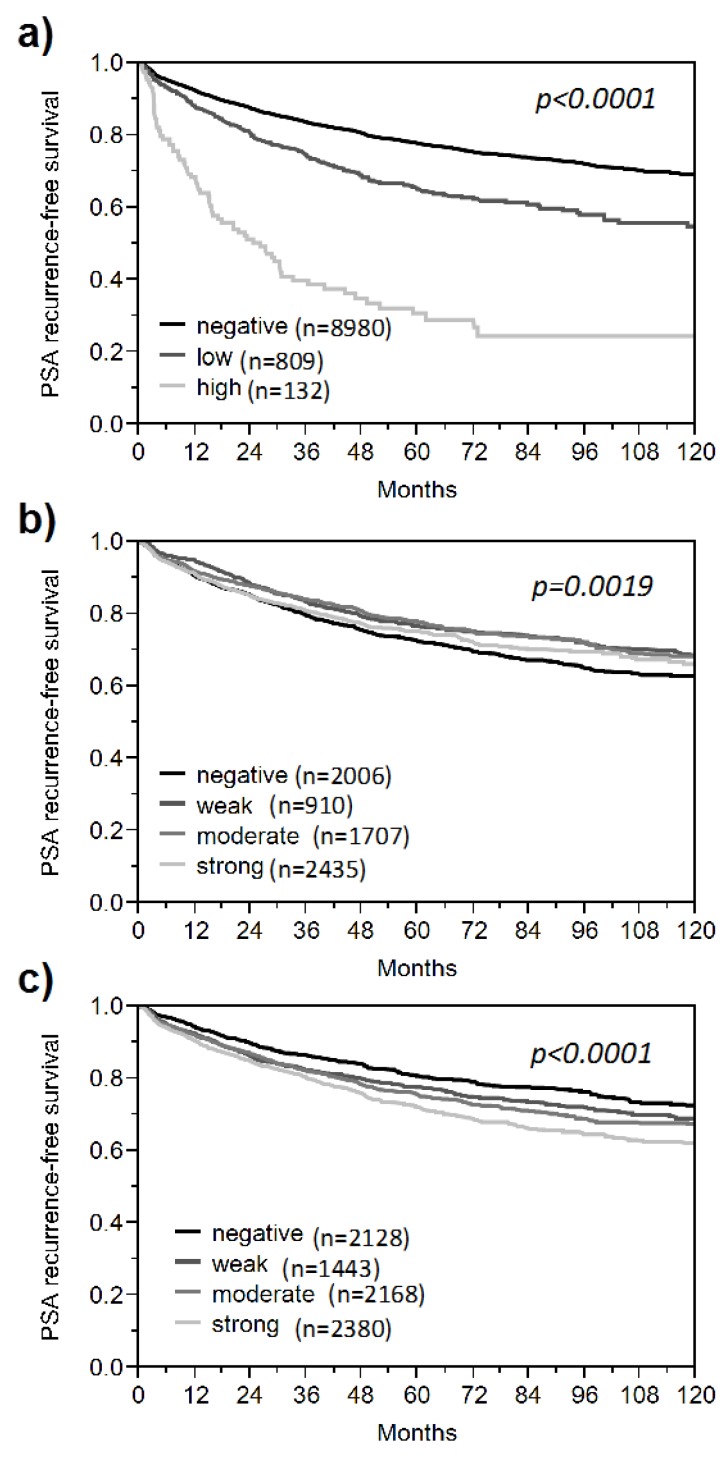
Examples of prognosis markers in prostate cancer. Kaplan Meier plots using biochemical recurrence as a clinical endpoint to demonstrate the clinical impact of (**a**) p53 as an example of a strong marker, (**b**) cluster of differentiation 147 (CD147) as an example of a very weak marker, and (**c**) LPCAT as an example of a moderate marker of prognosis.

**TMA Database.** The molecular database attached to this TMA contained results on more than 100 molecular markers. For example, we analyzed expression of therapy target genes like epidermal growth factor receptor (EGFR) [25] and human epidermal growth factor receptor 2 (HER2) [26], putative prognosticators including p53 [7,27], proliferation marker Ki67 [5], mammalian target of rapamycin (mTOR) [28], cluster of differentiation (CD) 10 [29], serine peptidase inhibitor Kazal type 1 (SPINK1) [30], karyopherin alpha 2 (KPNA2) [31], cysteine-rich secretory protein 3 (CRISP3) [32], nibrin (NBS1) [33], RNA binding motif protein 3 (RMB3) [34], and lysophosphatidylcholine acyltransferase 1 (LPCAT1) [35], mitochondrial content [36], prostate-specific markers like prostate specific antigen (PSA), prostate specific membrane antigen (PSMA) [37], alpha-methylacyl-CoA racemase (AMACR), and androgen receptor (AR), microvessel density [38], or immunological target proteins like CD117 [39], CD147 [40], and CD151 [41], and determined gene copy number alterations of important tumor suppressor loci in prostate cancer, including 8p (lipoprotein lipase, LPL) [42], 3p13 (forkhead box P1, FOXP1) [43], 5q21 (chromodomain helicase DNA binding protein 1, CHD1) [44], 6q15 (mitogen-activated protein kinase kinase kinase 7, MAP3K7) [45], 10q23 (PTEN) [46], TMPRSS2:ERG fusion [47] and PTEN breakage [48]. For this study, we selected 39 different protein markers that predicted patient prognosis if analyzed in our current 12,427 samples TMA or in an earlier version of the TMA comprising 11,156 of the 12,427 samples. Based on the results of previous survival analyses using biochemical (PSA) recurrence as a clinical endpoint, our selection included very strong prognostic markers (e.g., the p53 tumor suppressor, Figure 1a [27]), markers with only marginal prognostic relevance (e.g., CD147, Figure 1b [40]), and those with an intermediate prognostic impact (e.g., LPCAT1, Figure 1c [35]). 

**Immunohistochemistry (IHC).** Freshly cut TMA sections were used for all experiments. IHC analysis was performed using an ETS-related gene (ERG)-specific antibody as described before [47] and an antibody directed against vimentin to identify tissue samples that might have impaired immunoreactivity. For vimentin detection, freshly cut TMA sections were immunostained on one day and in one experiment. Slides were deparaffinized and exposed to heat-induced antigen retrieval for 5 minutes at 100 °C in pH 6 Tris-EDTA-Citrate buffer. Primary antibody specific for vimentin (mouse monoclonal antibody, DAKO, Glostrup, DK; clone V9; dilution 1:18,000) was applied at 37 °C for 60 minutes. Bound antibody was then visualized using the EnVision Kit (Dako, Glostrup, Denmark) according to the manufacturer’s directions. Presence or absence of ERG and vimentin staining was recorded in all tissue spots.

**Table 1:** Composition of the prognosis tissue microarray containing 12,427 prostate cancer specimens. The percentage in the column “Study cohort on tissue microarray (TMA)” refers to the fraction of samples across each category. The percentage in column “Biochemical relapse among categories” refers to the fraction of samples with biochemical relapse within each parameter in the different categories. pT, pathological tumor stage; pN, pathological lymph node stage.

Parameter	No. of patients (%)	
	Study cohort on TMA	Biochemical relapse among categories
	(n = 12,427)	
**Follow-up (mo)**		
n	11,665 (93.9%)	2769 (23.7%)
Mean	48.9	-
Median	36.4	-
Age (y)		
≤50	334 (2.7%)	81 (24.3%)
51–59	3061 (24.8%)	705 (23%)
60–69	7188 (58.2%)	1610 (22.4%)
≥70	1761 (14.3%)	370 (21%)
**Pretreatment** prostate specific antigen **(PSA) (ng/mL)**		
<4	1585 (12.9%)	242 (15.3%)
4–10	7480 (60.9%)	1355 (18.1%)
10–20	2412 (19.6%)	737 (30.6%)
>20	812 (6.6%)	397 (48.9%)
**pT category (AJCC 2002)**		
pT2	8187 (66.2%)	1095 (13.4%)
pT3a	2660 (21.5%)	817 (30.7%)
pT3b	1465 (11.8%)	796 (54.3%)
pT4	63 (0.5%)	51 (81%)
**Gleason grade**		
≤3 + 3	2983 (24.1%)	368 (12.3%)
3 + 4	6945 (56.2%)	1289 (18.6%)
4 + 3	1848 (15%)	788 (42.6%)
≥4 + 4	584 (4.7%)	311 (53.3%)
**pN category**		
pN0	6970 (91%)	1636 (23.5%)
pN+	693 (9%)	393 (56.7%)
**Surgical margin**		
Negative	9990 (81.9%)	1848 (18.5%)
Positive	2211 (18.1%)	853 (38.6%)

Numbers do not always add up to 12,427 in the different categories because of cases with missing data. Abbreviation: AJCC, American Joint Committee on Cancer.

**Fluorescence *in situ* hybridization (FISH).** A 4-μm TMA section was used for two-color FISH. For proteolytic slide pretreatment, a commercial kit was used (Paraffin pretreatment reagent kit, Vysis). A Spectrum-Orange–labeled HER2 probe was used together with a Spectrum-Green–labeled centromere 17 probe (PathVysion; Abbott Molecular). Before hybridization, TMA sections were de-paraffinized, air dried, and dehydrated in 70%, 85%, and 100% ethanol followed by denaturation for 5 minutes at 74 °C in 70% formamide-2× SSC solution. Following overnight hybridization at 37 °C in a humidified chamber, slides were washed and counterstained with 0.2 μmol/L 4',6-diamidino-2-phenylindole, an antifade solution. Presence or absence of red and green FISH signals was recorded in all tissue spots.

**Statistics**. Statistical calculations were performed with JPM 9 (JMP^®^, Version 9. SAS Institute Inc., Cary, NC, USA, 1989–2007) Contingency tables and the *chi²*-test were performed to search for associations between molecular parameters and tumor phenotype. Survival curves were calculated according to Kaplan‑Meier. The Log-Rank test was applied to detect significant differences between groups. 

## 3. Results and Discussion

### 3.1. Impact of the Tissue Quality

**Figure 2:**
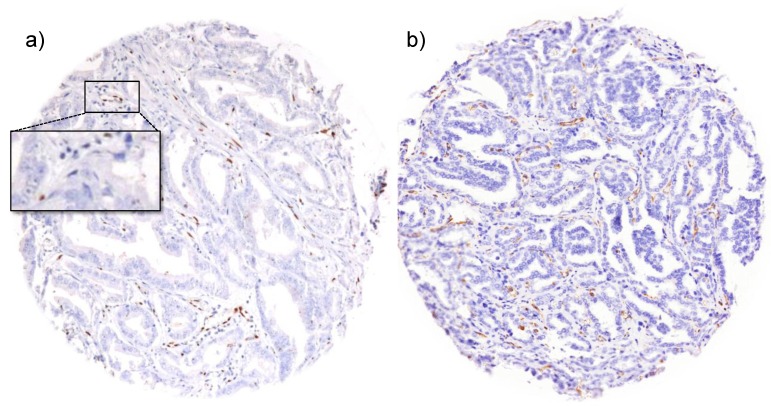
Examples of immunostainings of markers for tissue quality. (**a**) ETS-related gene (ERG) expression in endothelial cells and lymphocytes in a prostate cancer tissue spot. Tumor cells are ERG negative. The inset shows magnification of a blood vessel. (**b**) Vimentin expression in mesenchymal cells in a prostate cancer tissue spot. Tumor cells are vimentin negative.

In order to identify tissues with poor immunoreactivity, we performed ERG and vimentin immunohistochemistry analysis of our TMA. These proteins are expressed in virtually every human tissue. ERG is a member of the E26 transformation-specific (ETS) transcription factor family that is expressed in endothelial cells. ERG had been extensively studied in our TMA before since it is strongly expressed in about 50% of prostate cancers [47], and has been linked to early onset prostate cancer [48]. For the purpose of identifying low quality tissues, we re-analyzed our large 12,427 samples prostate cancer TMA for ERG expression specifically in endothelial cells (Figure 2a). In addition, we stained the TMA for vimentin, a type III intermediate filament that is strongly expressed in mesenchymal cells, which typically accompany prostate cancer cells (Figure 2b). Since blood vessels and mesenchymal cells can be found in virtually every prostate tissue sample, we considered complete absence of vimentin and ERG staining as an indicator of impaired immunoreactivity. In addition, we took advantage of the results from an earlier study, where we demonstrated that low-quality tissues with impaired immunoreactivity also showed a poor performance in fluorescence *in-situ* hybridization (FISH) analysis of gene copy numbers [49]. In the present study, we performed HER2 FISH analysis on the TMA and considered absence of FISH signals as an additional indicator of poor tissue quality. In summary, all tissue spots that showed simultaneous lack of ERG and vimentin immunostaining and absence of HER2 FISH signals were considered “low-quality”. 

**Figure 3:**
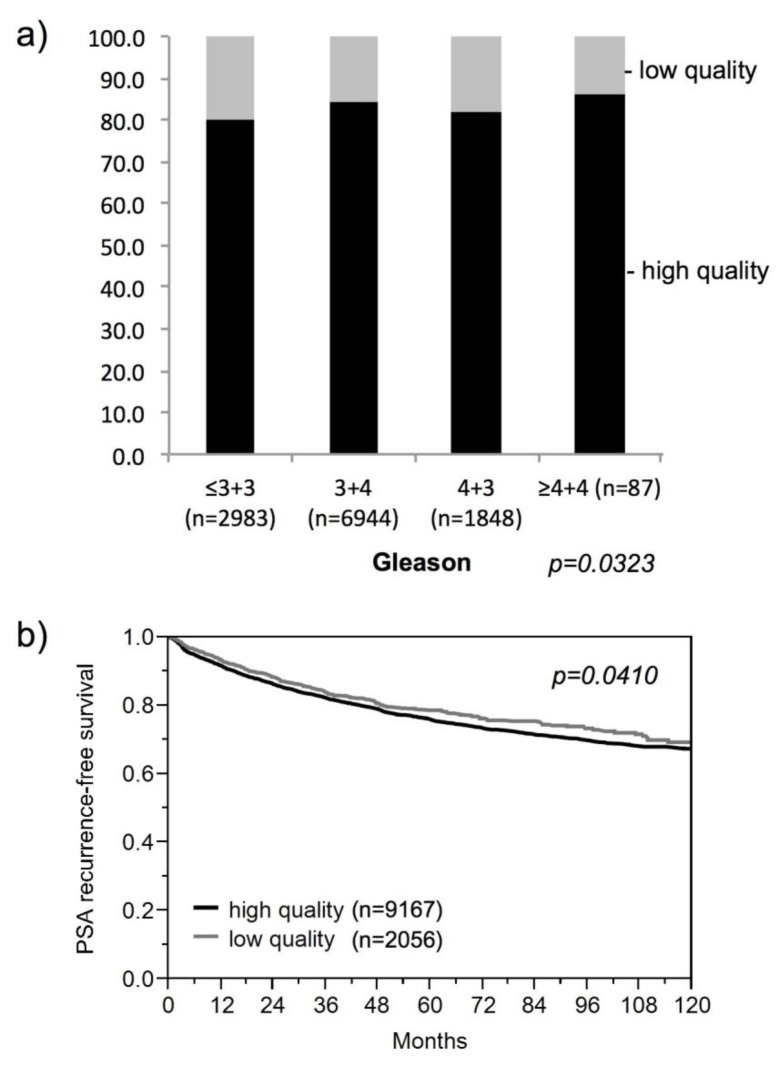
Lack of relevant associations between tissue quality and prostate cancer phenotype. (**a**) Relationship with the Gleason grade. (**b**) Relationship with biochemical recurrence.

A total of 11,223 tissue spots was included in this analysis. The remaining tissue spots were excluded because they were severely damaged or absent in the TMA slides. Simultaneous lack of ERG, vimentin, and HER2 signals were found in 2056 (18.3%) of the analyzed tissue spots. These “low-quality” tissues were randomly distributed across the TMA, and there was no obvious association between tissue reactivity and tumor phenotype or patient outcome (Figure 3). The marginally significant *p*-values obtained in these analyses do not reflect true associations but are attributable to slight variations between the groups.

**Figure 4:**
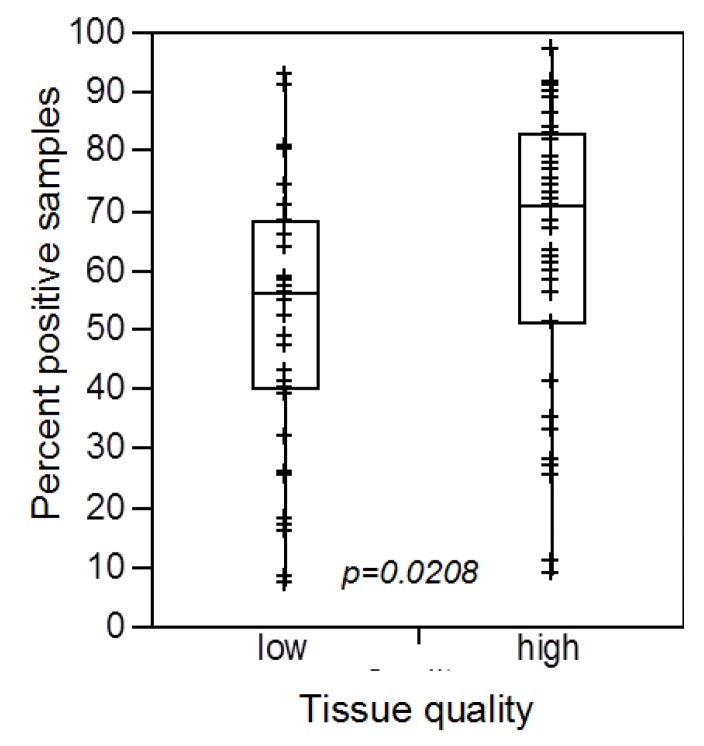
Impact of the tissue quality of the overall fraction of samples yielding a positive result in immunohistochemistry studies.

To further investigate the performance of these “low-quality” spots in IHC experiments, we next compared them to staining patterns of our 39 IHC markers in subsets of 2000 “low-quality” and 2000 “high-quality” tissues (*i.e.*, samples that were positive for all of ERG, vimentin and HER2). This analysis revealed that, although the “low-quality” tissues can be stained with most of the tested antibodies, there was a average reduction of about 12 percent points in the fraction of positive tissue samples across all of these markers in “low-quality” tissues as compared to “high-quality” tissues (Figure 4). These date demonstrate, that “low-quality” tissues bear a high risk to underestimate the true expression level and may even result in false negative findings.

**Figure 5:**
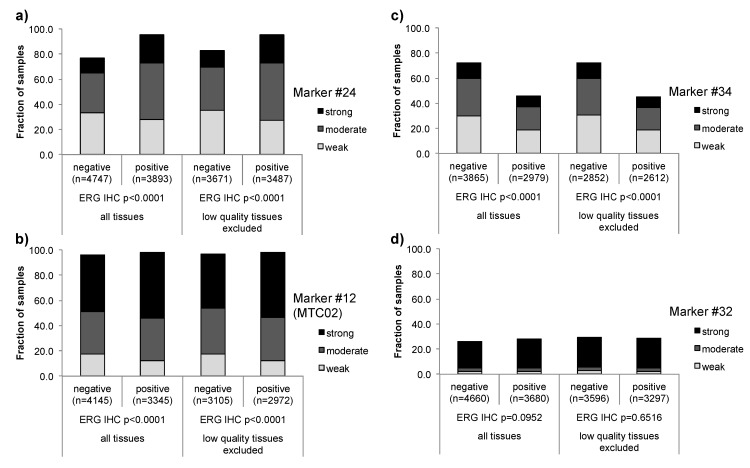
Examples of associations between expression of ERG and other Immunohistochemistry (IHC) markers in all tissue samples included in the 12,247 prostate cancers TMA (all tissues) and after exclusion of “low-quality” tissues from the analysis. (**a**) strong positive association, (**b**) weak positive association, (**c**) strong inverse association, (**d**) no association.

These findings imply that problems may arise if it comes to comparisons between biomarkers analyzed by immunohistochemistry. In such a scenario, false positive associations can potentially occur if the level of immunostaining of the analyzed markers parallels the quality of the tissue in a relevant fraction of samples. In contrast, inverse associations must always be considered valid. Here, the same tissues that are negative for one marker stain positive for the other, thus excluding the possibility of false associations due to reduced immunoreactivity. To assess the potential impact of “low-quality” tissues on the reliability of associations between ERG and other IHC markers, we used our existing ERG IHC data [47], which showed a positive result in about 50% of cancers. We studied the associations of all 39 markers to ERG expression, including markers with strong associations to ERG positivity (e.g., Marker #24, Figure 5a), markers with strong associations to ERG negativity (e.g., Marker #34, Figure 5c), markers with weak associations to ERG positivity (e.g., #12 (MTC02), Figure 5b), and markers lacking such associations (e.g., Marker #32, Figure 5d). Particularly for the latter set of markers, it could be possible that “low-quality” tissues drive such weak associations. All analyses were performed in differently sized subsets of our large TMA, and the significance of associations was compared between tissue sets containing both “low” and “high”‑quality tissues and tissue sets after excluding the 2056 “low-quality” tissues from the data. Since statistical associations will become stronger the more samples are analyzed, we performed the analyses in randomly selected subsets of 1600, 3200, 6400, and 10,000 samples. To compensate for incidental findings that might arise from random subset selection, we repeated each analysis five times. The Log-rank *chi^2 ^p*-value was recorded from each analysis, and the average Log‑rank *chi^2 ^p*-value was calculated from the five repeated analyses. All results are shown in Table 2. Following the same analysis strategy, we also questioned whether the reduced immunoreactivity in the “low-quality” tissues impacted the outcome of prognosis associations. For this analysis, we selected five of our 39 prognostic markers set and performed Kaplan-Meier survival plots to compare the impact of these markers (using biochemical (PSA) recurrence as clinical endpoint) before and after exclusion of the 2056 “low-quality” tissues. All results are shown in Table 3, and examples of Kaplan-Meier plots are given in Figure 4.

In both sets of calculations, we did not observe changes in the analysis results, regardless if the “low-quality” tissue was excluded from the analysis or not, demonstrating that the ≈20% “low-quality” tissues present in our TMA did not significantly impact the study outcome. Nevertheless, the examples of associations with ERG expression given in Figure 5 confirm that the fraction of entirely negative samples can be slightly overestimated unless the “low-quality” tissues are exclude, as indicated by a difference of five percent points between tumors with a negative result for both Marker #24 and ERG in subsets of cancers before and after exclusion of the “low-quality” tissues. However, the finding that even positive associations resulting from discrete expression differences remained significant after exclusion of the “low-quality” tissues (Figure 5b) clearly demonstrates that associations between different markers can be reliably detected in large-scale TMA studies. Since “low-quality” tissues were randomly distributed across all samples irrespective of the clinical course (Figure 3), it was not surprising that there was no difference in the ability to detect prognostic differences in tissues with or without “low-quality” tissues. Here, the “underestimation” of the true staining intensity resulted in a smooth shift of all survival curves either towards an overall better prognosis (*i.e.*, if strong expression of the marker was linked to poor prognosis), or towards worse prognosis (*i.e.*, if strong expression of the marker was linked to good prognosis), whereas the relative distance between the curves remained largely constant. However, this analysis also suggested that the number of samples included in marker validation analyses might have a much stronger impact on the analysis result than the tissue quality, thus prompting us to analyze the impact of the sample size in more detail below. 

**Table 2:** Impact of the tissue quality on the association between expression of ERG and other IHC markers. The *chi^2 ^p*-values are given for survival analyses in subsets of 1600–10,000 tissue spots. “Low quality tissue” indicates whether tissues with impaired immunoreactivity were excluded from analysis or not (included). “Association strength” separates the markers into those with weak, moderate, or strong positive associations (*i.e.*, the marker is more frequently expressed in ERG positive than in ERG negative cancers), those with inverse associations (*i.e.*, the marker is more frequently expressed in ERG negative than in ERG positive cancers), and those that are unrelated to ERG (no association).

Marker	Low quality tissue	Number of analyzed tissue spots				Association strength
		1600	3200	6400	10,000	
Marker #32	included	*0.1039*	*0.5243*	*0.1879*	*0.0952*	***No association***
	excluded	*0.9431*	*0.6612*	*0.7653*	*0.6515*	
Marker #13	included	*0.0112*	*<0.0001*	*<0.0001*	*<0.0001*	***Weak***
	excluded	*0.012*	*0.0004*	*<0.0001*	*<0.0001*	
Marker #12 (MTC02)	included	*<0.0001*	*<0.0001*	*<0.0001*	*<0.0001*	***Weak***
	excluded	*0.0238*	*<0.0001*	*<0.0001*	*<0.0001*	
Marker #31	included	*0.1307*	*0.0091*	*<0.0001*	*<0.0001*	***Weak***
	excluded	*0.2414*	*0.0031*	*<0.0001*	*<0.0001*	
Marker #37	included	*0.0017*	*<0.0001*	*<0.0001*	*<0.0001*	***Weak***
	excluded	*0.1159*	*0.0012*	*0.0008*	*<0.0001*	
Marker #7	included	*<0.0001*	*<0.0001*	*<0.0001*	*<0.0001*	***Moderate***
	excluded	*<0.0001*	*<0.0001*	*<0.0001*	*<0.0001*	
Marker #10	included	*<0.0001*	*<0.0001*	*<0.0001*	*<0.0001*	***Moderate***
	excluded	*<0.0001*	*<0.0001*	*<0.0001*	*<0.0001*	
Marker #2 (CD10)	included	*<0.0001*	*<0.0001*	*<0.0001*	*<0.0001*	***Moderate***
	excluded	*<0.0001*	*<0.0001*	*<0.0001*	*<0.0001*	
Marker #21	included	*<0.0001*	*<0.0001*	*<0.0001*	*<0.0001*	***Moderate***
	excluded	*<0.0001*	*<0.0001*	*<0.0001*	*<0.0001*	
Marker #27	included	*<0.0001*	*<0.0001*	*<0.0001*	*<0.0001*	***Moderate***
	excluded	*<0.0001*	*<0.0001*	*<0.0001*	*<0.0001*	
Marker #39 (p53)	included	*<0.0001*	*<0.0001*	*<0.0001*	*<0.0001*	***Moderate***
	excluded	*<0.0001*	*<0.0001*	*<0.0001*	*<0.0001*	
Marker #4	included	*<0.0001*	*<0.0001*	*<0.0001*	*<0.0001*	***Moderate***
	excluded	*<0.0001*	*<0.0001*	*<0.0001*	*<0.0001*	
Marker#3	included	*<0.0001*	*<0.0001*	*<0.0001*	*<0.0001*	***Moderate***
	excluded	*<0.0001*	*<0.0001*	*<0.0001*	*<0.0001*	
Marker #5	included	*<0.0001*	*<0.0001*	*<0.0001*	*<0.0001*	***Moderate***
	excluded	*<0.0001*	*<0.0001*	*<0.0001*	*<0.0001*	
Marker #33	included	*<0.0001*	*<0.0001*	*<0.0001*	*<0.0001*	***Moderate***
	excluded	*<0.0001*	*<0.0001*	*<0.0001*	*<0.0001*	
Marker #16 (NBS1)	included	*<0.0001*	*<0.0001*	*<0.0001*	*<0.0001*	***Moderate***
	excluded	*<0.0001*	*<0.0001*	*<0.0001*	*<0.0001*	
Marker #18 (AR)	included	*<0.0001*	*<0.0001*	*<0.0001*	*<0.0001*	***Moderate***
	excluded	*<0.0001*	*<0.0001*	*<0.0001*	*<0.0001*	
Marker #22	included	*<0.0001*	*<0.0001*	*<0.0001*	*<0.0001*	***Moderate***
	excluded	*<0.0001*	*<0.0001*	*<0.0001*	*<0.0001*	
Marker #24	included	*<0.0001*	*<0.0001*	*<0.0001*	*<0.0001*	***Moderate***
	excluded	*<0.0001*	*<0.0001*	*<0.0001*	*<0.0001*	
Marker #23	included	*<0.0001*	*<0.0001*	*<0.0001*	*<0.0001*	***Moderate***
	excluded	*<0.0001*	*<0.0001*	*<0.0001*	*<0.0001*	
Marker #36 (KPNA2)	included	*<0.0001*	*<0.0001*	*<0.0001*	*<0.0001*	***Moderate***
	excluded	*<0.0001*	*<0.0001*	*<0.0001*	*<0.0001*	
Marker #35	included	*<0.0001*	*<0.0001*	*<0.0001*	*<0.0001*	***Moderate***
	excluded	*<0.0001*	*<0.0001*	*<0.0001*	*<0.0001*	
Marker #30	included	*<0.0001*	*<0.0001*	*<0.0001*	*<0.0001*	***Moderate***
	excluded	*<0.0001*	*<0.0001*	*<0.0001*	*<0.0001*	
Marker #6 (FOXP2)	included	*<0.0001*	*<0.0001*	*<0.0001*	*<0.0001*	***Strong***
	excluded	*<0.0001*	*<0.0001*	*<0.0001*	*<0.0001*	
Marker #8	included	*<0.0001*	*<0.0001*	*<0.0001*	*<0.0001*	***Strong***
	excluded	*<0.0001*	*<0.0001*	*<0.0001*	*<0.0001*	
Marker #9	included	*<0.0001*	*<0.0001*	*<0.0001*	*<0.0001*	***Strong***
	excluded	*<0.0001*	*<0.0001*	*<0.0001*	*<0.0001*	
Marker #11	included	*<0.0001*	*<0.0001*	*<0.0001*	*<0.0001*	***Strong***
	excluded	*<0.0001*	*<0.0001*	*<0.0001*	*<0.0001*	
Marker #14	included	*<0.0001*	*<0.0001*	*<0.0001*	*<0.0001*	***Strong***
	excluded	*<0.0001*	*<0.0001*	*<0.0001*	*<0.0001*	
Marker #15 (LPCAT)	included	*<0.0001*	*<0.0001*	*<0.0001*	*<0.0001*	***Strong***
	excluded	*<0.0001*	*<0.0001*	*<0.0001*	*<0.0001*	
Marker #19 (RBM3)	included	*<0.0001*	*<0.0001*	*<0.0001*	*<0.0001*	***Strong***
	excluded	*<0.0001*	*<0.0001*	*<0.0001*	*<0.0001*	
Marker #26	included	*<0.0001*	*<0.0001*	*<0.0001*	*<0.0001*	***Strong***
	excluded	*<0.0001*	*<0.0001*	*<0.0001*	*<0.0001*	
Marker #26	included	*<0.0001*	*<0.0001*	*<0.0001*	*<0.0001*	***Strong***
	excluded	*<0.0001*	*<0.0001*	*<0.0001*	*<0.0001*	
Marker #25	included	*<0.0001*	*<0.0001*	*<0.0001*	*<0.0001*	***Strong***
	excluded	*<0.0001*	*<0.0001*	*<0.0001*	*<0.0001*	
Marker #29	included	*<0.0001*	*<0.0001*	*<0.0001*	*<0.0001*	***Strong***
	excluded	*<0.0001*	*<0.0001*	*<0.0001*	*<0.0001*	
Marker #1 (CD147)	included	*<0.0001*	*<0.0001*	*<0.0001*	*<0.0001*	***Inverse***
	excluded	*<0.0001*	*<0.0001*	*<0.0001*	*<0.0001*	
Marker #17	included	*<0.0001*	*<0.0001*	*<0.0001*	*<0.0001*	***Inverse***
	excluded	*<0.0001*	*<0.0001*	*<0.0001*	*<0.0001*	
Marker #20	included	*<0.0001*	*<0.0001*	*<0.0001*	*<0.0001*	***Inverse***
	excluded	*0.0004*	*<0.0001*	*<0.0001*	*<0.0001*	
Marker #34	included	*<0.0001*	*<0.0001*	*<0.0001*	*<0.0001*	***Inverse***
	excluded	*<0.0001*	*<0.0001*	*<0.0001*	*<0.0001*	
Marker #38	included	*<0.0001*	*<0.0001*	*<0.0001*	*<0.0001*	***Inverse***
	excluded	*<0.0001*	*<0.0001*	*<0.0001*	*<0.0001*	

**Table 3:** Impact of the tissue quality on the outcome of Kaplan-Meier survival analysis. The *chi^2^p*-values are given for survival analyses in subsets of 1600–10,000 tissue spots. “Low quality tissue” indicates whether tissues with impaired immunoreactivity were excluded from analysis or not (included).

Marker	Low quality tissue	Number of analyzed tissue spots			
		1600	3200	6400	10,000
#2 (CD10)	included	*0.0937*	*0.0006*	*<0.0001*	*<0.0001*
	excluded	*0.1215*	*0.0005*	*<0.0001*	*<0.0001*
#3	included	*0.0761*	*0.0946*	*0.0595*	*0.0037*
	excluded	*0.1146*	*0.0761*	*0.0385*	*0.0043*
#4	included	*0.0810*	*0.1082*	*0.0151*	*<0.0001*
	excluded	*0.1059*	*0.0810*	*0.0060*	*<0.0001*
#18 (AR)	included	*0.0082*	*0.0206*	*0.0006*	*<0.0001*
	excluded	*0.0197*	*0.0082*	*0.0003*	*<0.0001*
#35	included	*<0.0001*	*<0.0001*	*<0.0001*	*<0.0001*
	excluded	*0.0230*	*<0.0001*	*<0.0001*	*<0.0001*

### 3.2. Impact of the Sample Size

**Figure 6:**
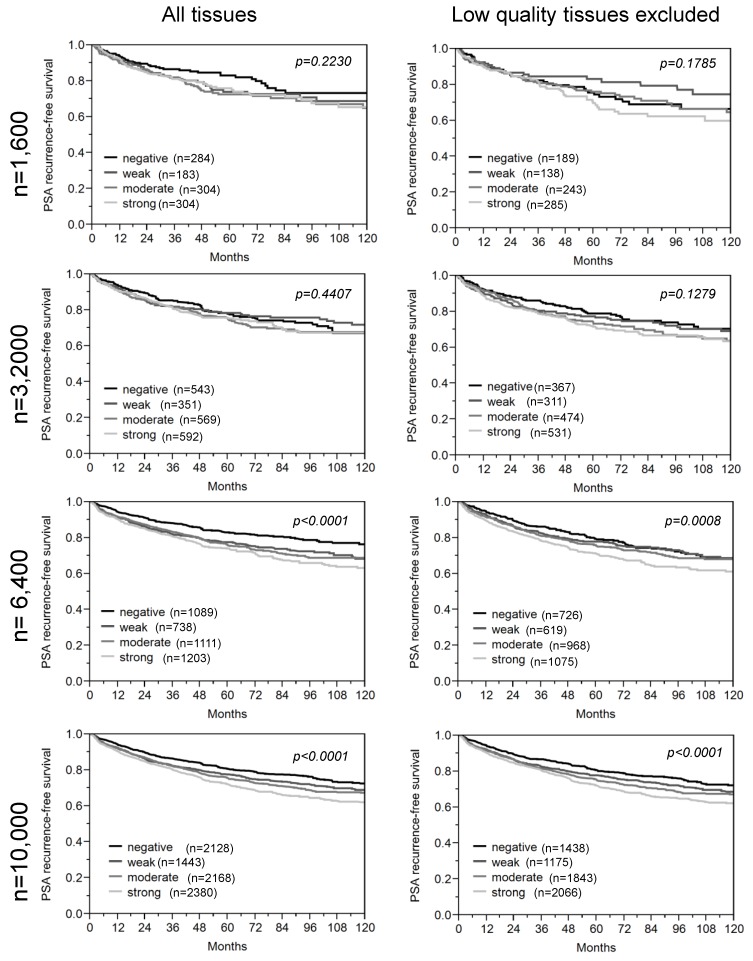
Examples of Kaplan-Meier plots obtained from Marker #4 in subsets of 1600–10,000 samples. “All tissues” indicates that the samples contain low-quality and high-quality samples.

In order to estimate the minimal sample size that is required to yield statistically stable results in prostate cancer prognosis marker validation studies, we carried out serial analyses in randomly selected subsets of 50, 100, 200, 400, 800, 1600, 3200, 6400 and all (12,427) samples included in our TMA. We performed Kaplan-Meier survival plots and Log-rank *chi^2^* tests including a total of 39 protein markers with confirmed prognostic relevance from our molecular database. The smallest sample set that revealed a Log-rank *p*-value of 0.001 or less was considered to be sufficient for reliable marker analysis provided that this significance level held also true in the analysis of all larger sample sets. In addition, in order to rank the “prognostic power” of our 39 markers, we summarized the Log-rank values emerging from all subset analyses of each marker. This strategy was selected because *chi^2^* values can be easily extracted from all tests and thus provide a simple, however objective, index of the power of individual markers. We grouped our markers according to the accumulated *chi^2^* values into markers with “weak” (sum of all *chi^2^* values <100), “moderate” (sum *chi^2^* 101–299), and “strong” prognostic power (sum *chi^2^* ≥300). The Log-rank *p*-values for all markers in each sample subset and the accumulated Log-rank *chi^2^* values per marker are shown in Table 4, and exemplary Kaplan-Meier plots are given in Figure 6. 

**Figure 7:**
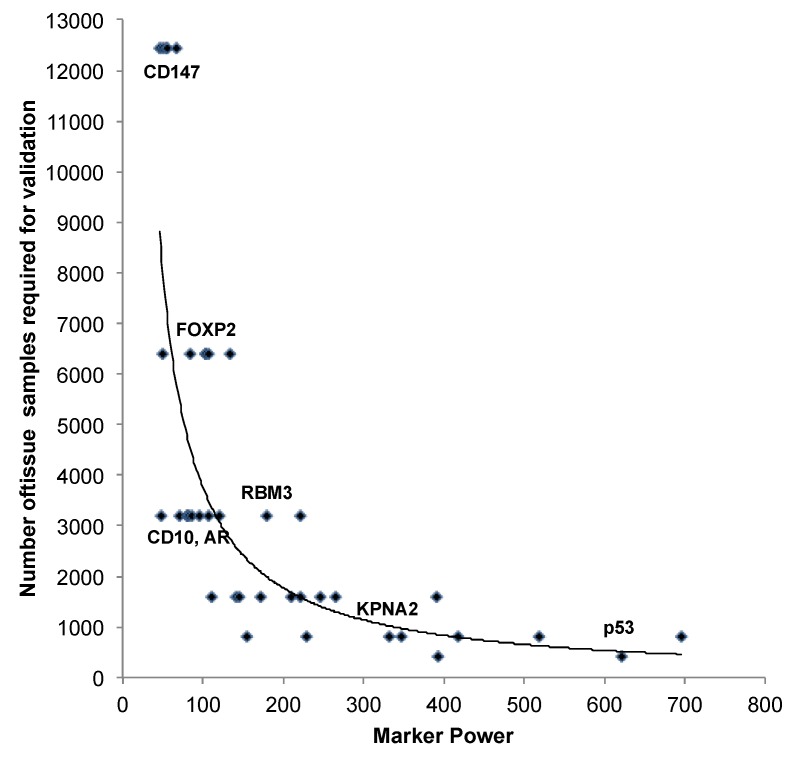
Association between the “prognostic power” of different immunohistochemistry markers and the minimal number of samples that is required for statistically sound marker validation studies. “Marker Power” is given as the sum of Log-rank *chi^2^* values per marker from the analysis in subsets of 50, 100, 200, 400, 800, 1600, 3200, 6400, and 12,427 samples. Some markers are annotated as examples.

The results of this analysis first of all demonstrate a close relationship between the “prognostic power” of a marker and the numbers of samples that need to be analyzed in order to reliably evaluate the marker’s prognostic potential (Figure 7 and Table 4). Given that the power of a marker of interest is typically not known before the analysis is performed (particularly in case of novel and uncharacterized candidate markers), and that four markers revealed prognostic relevance only if the entire sample set was analyzed (Table 4), our findings imply that as many samples as possible should be included in such marker validation experiments in order to also reliably detect minor associations between prostate cancer genotype and clinical behavior. However, from a more practical point of view, our data also demonstrates that a cohort size of 6400 prostate cancers is sufficient to reproduce the prognostic value of the vast majority (*i.e.*, 35 out of 39, 90%) of the markers included in our study. 

**Table 4:** Impact of the sample size on the outcome of Kaplan-Meier survival analyses. The *chi^2^p*-values are given for survival analyses in subsets of 50–12,427 tissue spots. “n analyzable” gives the number of interpretable tissue spots if the entire TMA was analyzed. “Marker power” indicates the relative prognostic power as described in the results section. Bold face indicates the minimal sample set that was considered sufficient to evaluate the respective marker. Grey color indicates sample sizes that yielded strong prognostic relevance.

Marker	n analy-zable	Number of analyzed tissue spots									Marker Power
		50	100	200	400	800	1600	3200	6400	12,427	
Marker #1 (CD147)	7605	0.2417	0.4218	0.4550	0.4984	0.5396	0.3539	0.3428	0.0379	**0.0019**	weak
Marker #4	8025	0.0863	0.0998	0.9252	0.4330	0.4648	0.5781	0.0571	0.0666	**0.0002**	weak
Marker #3	7574	0.2276	0.0720	0.0673	0.7038	0.6825	0.0934	0.3195	0.0351	**0.0016**	weak
Marker #5	9473	0.8553	0.2279	0.9171	0.5532	0.1026	0.3192	0.0797	**0.0097**	0.0016	weak
Marker #6 (FOXP2)	8284	0.7963	0.4436	0.0082	0.4058	0.4309	0.0776	0.0308	**0.0011**	<0.0001	weak
Marker #7	9485	<0.0001	0.4999	0.0056	0.2196	0.0492	0.0464	0.0796	**0.0007**	<0.0001	moderate
Marker #8	8158	0.1175	0.5066	0.7638	0.1192	0.3055	0.0020	0.0548	**<0.0001**	<0.0001	moderate
Marker #9	6494	0.0494	0.9606	0.5709	0.1780	0.0603	0.0421	0.0564	**<0.0001**	<0.0001	moderate
Marker #33	9262	0.1088	0.2166	0.5932	0.6114	0.0017	0.0013	0.0105	**<0.0001**	<0.0001	moderate
Marker #10	9516	0.6617	0.8651	0.0836	0.1074	0.1415	0.0489	**0.0041**	0.0034	<0.0001	weak
Marker #2 (CD10)	8488	0.0722	0.5977	0.6268	0.2465	0.3409	0.5137	**0.0001**	0.0062	0.0012	weak
Marker #11	9627	0.5518	0.1662	0.0652	0.4009	0.5480	0.1911	**0.0009**	0.0046	<0.0001	weak
Marker #12 (MTC02)	8407	0.4313	0.9545	0.7656	0.9315	0.3693	0.2517	**0.0013**	0.0004	<0.0001	weak
Marker #14	8654	0.7139	0.0146	0.6741	0.4165	0.1473	0.4119	**0.0094**	0.0002	<0.0001	weak
Marker #16 (NBS1)	8026	0.6506	0.0525	0.2071	0.7379	0.8382	0.2079	**0.0004**	0.0026	<0.0001	weak
Marker #13	9875	0.8336	0.3172	0.2557	0.6717	0.0790	0.0962	**0.0026**	0.0004	<0.0001	weak
Marker #15 (LPCAT)	8762	0.7141	0.1713	0.5933	0.6978	0.2428	0.0449	**0.0020**	0.0003	<0.0001	weak
Marker #17	7275	0.7259	0.2301	0.2169	0.3562	0.3492	0.0661	**0.0094**	<0.0001	<0.0001	moderate
Marker #18 (AR)	7856	0.4576	0.0246	0.9451	0.6439	0.7573	0.3058	**<0.0001**	0.0034	<0.0001	moderate
Marker #20	6638	0.7489	0.7507	0.2524	0.4930	0.9583	0.0863	**0.0001**	<0.0001	<0.0001	moderate
Marker #19 (RBM3)	8303	0.4712	0.1035	0.4893	0.2494	0.0134	0.0658	**0.0006**	<0.0001	<0.0001	moderate
Marker #21	9643	0.8921	0.6673	0.2718	0.0004	0.0086	0.0170	**<0.0001**	<0.0001	<0.0001	moderate
Marker #28	6824	0.5465	0.0252	0.8696	0.6919	0.2038	**0.0075**	0.0026	<0.0001	<0.0001	moderate
Marker #22	7117	0.2824	0.8367	0.5302	0.9432	0.0591	**0.0095**	<0.0001	<0.0001	<0.0001	moderate
Marker #24	9756	0.5724	0.3132	0.8763	0.6290	0.0686	**0.0016**	<0.0001	0.0002	<0.0001	moderate
Marker #23	9744	0.3087	0.0266	0.0008	0.1771	0.3495	**0.0001**	0.0004	<0.0001	<0.0001	moderate
Marker #29	9403	0.8852	0.0612	0.1260	0.1830	0.8574	**0.0028**	<0.0001	<0.0001	<0.0001	moderate
Marker #27	7588	0.0607	0.2364	0.2661	0.0050	0.4899	**0.0001**	<0.0001	<0.0001	<0.0001	moderate
Marker #26	9633	0.0169	0.5727	0.1760	0.7661	0.0491	**0.0003**	<0.0001	<0.0001	<0.0001	moderate
Marker #25	7677	0.8371	0.3814	0.7065	0.0233	0.9297	**<0.0001**	<0.0001	<0.0001	<0.0001	moderate
Marker #30	8174	0.3570	0.5619	0.3006	0.0028	0.1186	**0.0002**	<0.0001	<0.0001	<0.0001	strong
Marker #31	10215	0.4072	0.4062	0.2342	0.3143	**0.0040**	0.0023	0.0002	<0.0001	<0.0001	moderate
Marker #35	10216	0.8828	0.4813	0.1523	0.1304	**0.0018**	<0.0001	<0.0001	<0.0001	<0.0001	strong
Marker #34	7670	0.0833	0.3010	0.2137	0.0703	**0.0001**	0.0002	<0.0001	<0.0001	<0.0001	strong
Marker #37	7822	0.0044	0.0106	0.1209	0.0247	**0.0002**	<0.0001	<0.0001	<0.0001	<0.0001	strong
Marker #32	9467	0.8994	0.3867	0.0006	0.0640	**<0.0001**	0.0004	<0.0001	<0.0001	<0.0001	strong
Marker #36 (KPNA2)	7943	0.1490	0.3803	0.0483	**0.0044**	**<0.0001**	<0.0001	<0.0001	<0.0001	<0.0001	strong
Marker #39 (p53)	10946	0.2686	0.1962	0.0192	**<0.0001**	0.0055	<0.0001	<0.0001	<0.0001	<0.0001	strong
Marker #38	9576	0.7878	0.3611	**0.0091**	0.0063	<0.0001	<0.0001	<0.0001	<0.0001	<0.0001	strong

Importantly, only two (5%) of the 39 markers in our study, including p53 as a prime example of a very strong prognostic marker [27], had sufficient prognostic power to allow for conclusive results also in small cohorts including less than 500 prostate cancers. This finding is of particular interest since the majority of prostate cancer marker studies still analyze less than 500 cancer samples [5]. Our findings provide a simple explanation for the highly discrepant results on most potential prognostic biomarkers. We also found significant associations in less than 500 samples that, however, did not hold true in the next larger subsets and must, therefore, be considered incidental. For example, Marker #7 revealed significant *p*-values in subsets of 50 and 200 samples, but not in subsets of 100, 400, or even 3200 samples, demonstrating that analysis of small subsets can occasionally lead to incidental statistical results. 

**Figure 8:**
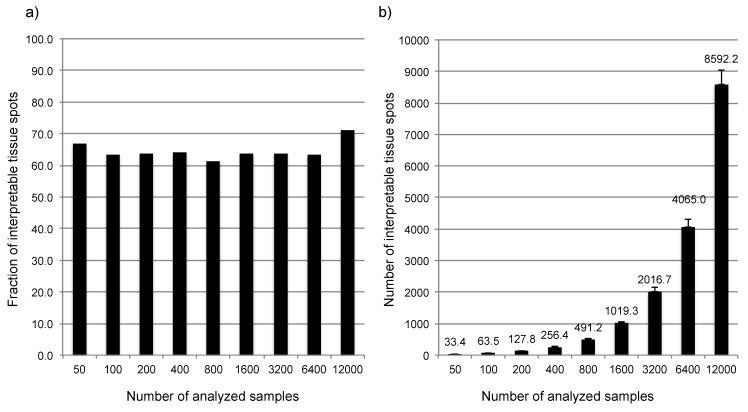
Association between the number of tissue samples analyzed in TMA studies and (**a**) the fraction or (**b**) total numbers of interpretable samples.

It is further of note that there is always a considerable fraction of samples that does not yield interpretable results. This is due to typical TMA-related issues, including exhausted tissue cores resulting in empty spots in the TMA section or lack of tumor cells in the tissue spot. In our study, the fraction of non-interpretable tissue cores was about 35%, independent from the size of the subset selected for analysis (Figure 8a). As a consequence, the number of interpretable samples varied between 6494 and 10,946 (average 8592) spots for the different markers in the entire dataset (n = 12,427) and averaged for example 33.4 cancers in the 50 samples subset, 1019.7 cancers in the 1600 samples subset, or 4065 cancers in the 6400 samples subset (Figure 8b). Therefore, a certain dropout rate of TMA spots should always be taken into account if a TMA is built. The fraction of interpretable samples can potentially be increased if multiple samples of the same cancer specimen are included in the TMA. We do not recommend this procedure, however. For example, building a 6000‑samples TMA from 2000 cancers with three spots from each cancer can be expected to result in about 1800 interpretable cancers (which still is too small a number for reliable statistical analysis according to our data), but is connected with the same costs, analysis time, and tissue consumption as compared to a 6000 samples TMA built from one punch per tumor, which will, however, result in about 3900 interpretable cancers. In addition, analysis of multiple cores always introduces a statistical bias into the analysis. This is because not all of the multiple tissue spots per tumor will be analyzable, and tumors with three to four interpretable spots might have a higher likelihood to detect positive staining as compared to tumors with only one to two interpretable tissue spots. 

## 4. Conclusions

The availability of a very large prostate cancer prognosis TMA with an extensive molecular database, including samples from more than 12,000 individual prostate cancers as well as molecular data from 39 prognostic relevant protein markers enabled us to evaluate the impact of qualitative and quantitative factors for prostate cancer biomarker studies. The results of our analyses suggest that such studies should aim at the analysis of at least 6000 individual prostate cancer samples to obtain reliable statistical findings allowing for a concluding judgment of a potential prognostic value of a marker of interest. Only for particularly strong markers, reliable results can also be obtained from substantially smaller cohorts. However, very strong prognostic markers appear to be rare, and the power of a marker is often not known before the analysis is made. Our data further suggest that almost 20% of the tissues included in a prostate cancer TMA may have limited tissue reactivity, potentially compromising the results of some analyses. While there is no impact of tissue reactivity on the results of prognostic studies, this issue is more relevant if it comes to comparisons between biomarkers analyzed by immunohistochemistry. Our data suggest, however, that even such associations that result from only discrete expression differences can be reliably identified in large-scale TMA analyses.
